# Real-Time Turning Movement, Queue Length, and Traffic Density Estimation and Prediction Using Vehicle Trajectory and Stationary Sensor Data

**DOI:** 10.3390/s25030830

**Published:** 2025-01-30

**Authors:** Amr K. Shafik, Hesham A. Rakha

**Affiliations:** Charles E. Via, Jr. Department of Civil and Environmental Engineering, Virginia Tech, Blacksburg, VA 24061, USA; ashafik@vt.edu

**Keywords:** turning movement counts, adaptive Kalman filters, queue estimation, connected vehicles, signal optimization

## Abstract

This paper introduces a two-stage adaptive Kalman filter algorithm to estimate and predict traffic states required for real-time traffic signal control. Leveraging probe vehicle trajectory and upstream detector data, turning movement (TM) counts in the vicinity of signalized intersections are estimated in the first stage, while the upstream approach density and queue sizes are estimated in the second stage. The proposed approach is evaluated using drone-collected and simulated data from a four-legged signalized intersection in Orlando, Florida. The performance of the two-stage approach is quantified relative to the baseline estimation without a Kalman filter. The results show that the Kalman filter is effective in enhancing traffic state estimates at various market penetration levels, where the filter both improves the estimation accuracy over the baseline case and provides reliable state predictions. In the first stage, the standard deviation (SD) in TM estimates improves by up to 50% compared to the estimates provided by the sole use of probe vehicle headings. The proposed approach also provides predictions with a minimal SD of 92.8 veh/h at a 5% level of market penetration. In the second stage, the proposed queue size estimation method results in an enhancement to the queue size estimation of up to 32.8% compared to the estimates obtained from the baseline approach. In addition, the estimated traffic density is enhanced by up to 18.5%. The proposed two-stage approach demonstrates the capability of providing reliable turning movement predictions across varying levels of market penetration. This highlights the readiness of this approach for practical application in real-time traffic signal control systems.

## 1. Introduction

Knowledge of turning movements (TMs) and queue sizes at signalized intersections is essential for numerous traffic applications, including conducting traffic analyses, traffic signal optimization, and evaluating the performance of the transportation system, especially for real-time applications. Specifically, predicting the traffic stream density and number of queued vehicles at signalized intersection approaches is critical for real-time adaptive traffic signal controllers. However, accurate TM counts and queues are hard to obtain because they require exhaustive data collection efforts.

Traditional methods for collecting traffic data at intersections, such as manual counts or fixed-point sensors, have long been the primary approach for gathering traffic state information. Manual observation requires field personnel to record vehicle movements and queues at intersections, but this process is labor-intensive, costly, and prone to human errors. Similarly, fixed-point sensors, such as loop detectors and video cameras, can automate data collection to some extent but come with their own set of challenges. Loop detectors are often limited to detecting vehicle presence without providing detailed information about individual vehicle trajectories, while video-based systems require advanced image processing algorithms to extract useful data. These methods also require costly installation and maintenance, and their accuracy can be affected by environmental factors such as weather conditions, lighting, and occlusions.

Recent advancements in connected vehicle (CV) technologies enable continuous traffic data collection through the communication between vehicles and infrastructure. This probe data, including vehicle positions, speeds, and headings, can significantly improve traffic state estimation, particularly for turning movements (TMs) and queue sizes at intersections [1]. Unlike traditional methods, CV data offers real-time availability, and higher granularity, and reduces the need for expensive infrastructure. While studies have shown that probe data can achieve high accuracy in estimating TMs and queues, challenges remain in fully realizing their potential.

A major challenge in using probe data for traffic state estimation is market penetration, as the effectiveness of CV-based data depends on the proportion of equipped vehicles. At low penetration levels, probe data becomes unreliable, necessitating robust estimation methods. Kalman filters, which combine real-time data with historical information, help improve accuracy and predict future traffic states. However, previous research efforts that utilized Kalman filters for traffic state estimation overlooked categorizing state estimates by turning movement, which is essential for some adaptive traffic signal controllers. Furthermore, most of the traffic state estimates are developed for highway traffic state estimation rather than in the vicinity of signalized intersections. In addition, the reported Kalman filter applications utilized historical data to calculate the covariance error parameters, where the availability of these data can be limited and the relevance to the time of application is questionable.

To address this need, this study proposes a two-stage estimation and prediction approach for real-time TMs, upstream link density, and the number of queued vehicles per direction at signalized intersections. The proposed approach provides horizon predictions, on a real-time basis, leveraging upstream loop detectors and probe vehicle data. The proposed adaptive Kalman filtering algorithm is designed to estimate and predict the real-time number of turning vehicles in the first stage, with the prediction horizon adaptable depending on the application’s requirements, which can be as short as 1, 5, and 10 s. Thus, the estimated TMs are utilized in the second stage for queue size and density estimation, which is conducted for each TM. The proposed approach provides the essential information that facilitates the field implementation of advanced traffic signal optimization systems, such as the game-theoretic traffic signal controller proposed by [2], which requires real-time predictions of TMs and queue size for each lane group.

## 2. Literature Review

### 2.1. Estimation of Turning Movement Counts

Numerous efforts in the literature have approached the turning movement estimation problem at signalized intersections using loop detector data. As the approaching traffic flow counts are relatively easy to obtain using detectors or other stationary sensors, multiple methods have been proposed in the literature to infer TMs using approaching flows. The manual trial-and-error technique is one of the methods that requires obtaining inflow and outflow traffic at all the approaches of the intersection. This approach is considered tedious and difficult in terms of mathematical calculations and efficiency [3]. Some studies presented more robust techniques to estimate the most likely origin–destination matrix for intersections, which best replicates the observed approach counts, such as the work of Hauer et al. [4], which estimated the matrix using a likelihood maximization approach using Kruithof’s algorithm. This technique showed acceptable estimation accuracy; however, prior knowledge about actual TMs is critical for performing this approach. In addition, this method requires the deployment of detectors in all directions that carry traffic to and from the intersection. Van Aerde et al. [5] developed a tool for estimating an origin–destination (OD) demand matrix. The tool utilizes a maximum-likelihood-based numerical solution that does not require flow continuity at the network nodes. It is noted that the origin–destination problem is under-specified, where multiple solutions will provide a match to the counts. The maximum likelihood is used to select the most likely solution out of these. A seed matrix can also be used to bias the solution towards the seed matrix.

More recent studies presented other methods to estimate TMs, such as the work of Xu et al. [6], which developed an automatic TM identification system (ATMIS) that estimates TMs on a real-time basis. Another study by Zhang et al. [7] estimated TMs using a nonlinear programming approach using inflow and outflow counts. This approach shows high consistency compared to actual TMs. However, these methods still require outflow detectors to obtain acceptable estimates.

Chen et al. [8] also used a nonlinear path flow-based algorithm to estimate TMs at a network level based on counts across the entire network. The study showed promising results in terms of estimation accuracy. However, the algorithm’s iterative process is considered difficult for real-time field applications in which TM estimations are required for short periods (e.g., 10–30 s).

Some studies used a genetic algorithm for TM estimations, such as the work of Jiao et al. [9], which estimated TMs in real-time by minimizing the deviations between observed and estimated traffic counts. Ghanim et al. [10] used artificial neural networks (ANNs) for the estimation. Results of the developed ANN model showed that ANNs can be used to accurately estimate TMs. Mousavizadeh et al. [11] utilized probe vehicle data for turning rate estimation at large-scale urban networks using a wavelet transform decomposition, using an estimation model that requires historic TM data. However, training machine learning models and training such estimation models based on historical data are considered computationally expensive and cannot be generalized beyond the context of the training data, such as the application to different characteristics of intersections, driving behavior, and demand patterns.

Some studies adopted a nonlinear least-square approach for the TM estimation problem such as the work of Lan et al. [12], which performed real-time TM estimation using partial counts on urban networks. The study employed a recursive nonlinear least-square approach using a partial set of detector counts. Similarly, Mirchandani et al. [13] presented a least-square minimization TM prediction algorithm implemented on a real-time basis. The algorithm is based on phase-to-phase counts that require the current turning proportions for prediction.

Finally, recent advancements in CV technologies have also been leveraged to estimate TMs, such as the work of Saldivar et al. [14], which used CV trajectory data to estimate TMs at signalized intersections. Entry and exit trajectory headings were used to detect the number of movement clusters using k-means clustering, which were assigned to a TM. A matching accuracy of up to 98% was achieved for over 1.1 million analyzed trajectories.

### 2.2. Queue Size Estimation from Probe Vehicles

The real-time queuing information at isolated intersections is essential information for the optimal control of traffic signals. The advancements in connected and automated vehicle technology allow the traffic signal controller to use probe vehicle data to make queue size estimates. There are vast contributions in the literature on the topic of queue estimation, which is mainly addressed in two primary estimation techniques as follows: the input-output method [15] and the shockwave theory. The input-output method estimates the number of vehicles within the queue, whereas the shockwave theory emphasizes the spatial extent of the queue. Furthermore, the queue estimation timeframe setting is categorized into a cycle-by-cycle queue length estimation and the real-time queue estimation [16].

Various methods of real-time queue estimation are reported in the literature such as the work of Comert et al. [17], which used a statistical method to estimate queue size in real-time from probe vehicle data. Based on the position of the probe vehicles in the queue, the queue size is estimated by a derived analytical expression. The study showed high estimation accuracy. However, it assumed that the marginal probability of queue size distribution is known, which is prior knowledge that is unavailable in many cases. Liu et al. [18] used a Markov model to estimate real-time queue size utilizing probe vehicle data. The average traffic flow rate, historical queue size data, and the stopping states for arriving probe vehicle data were used in the estimation procedure. The estimation scheme was tested for multiple market penetration levels of CVs. Results indicated high estimation accuracy and efficiency in handling the randomness in the system. One drawback is that the study relied on historical data, which might not be readily available. In addition, the categorization of queue size estimation by TM was also overlooked.

Another study by Zhao et al. [19] proposed a series of novel methods for real-time queue size estimation from probe vehicles. The proposed methods exploited the stopping positions of probe vehicles. The traffic volume and total queue size for each movement were obtained using the aggregated historical trajectory data. Study limitations include the inaccurate estimation of the market penetration rate using the last stopping position of probe vehicles. Shahrbabaki et al. [20] used a data fusion approach to estimate the second-by-second queue length. The proposed method showed notable estimation accuracy and efficiency.

Wei et al. [16] adopted an empirical Bayes method to estimate the cycle-based queue length, which showed significant estimation performance. Hao et al. [21] used a Bayesian network model for estimating cycle-by-cycle queue length. The study reported that 5% is the minimum market penetration rate for the practical application of queue estimation using probe vehicles. However, the cycle-to-cycle queue estimation methods are only considered applicable to conventional traffic signal controllers where the phase lengths are known. This is unlike other adaptive signals where the phase length is not known in advance.

### 2.3. Traffic State Estimation Using Kalman Filters

Kalman filters have been widely used in various traffic management and control applications. For instance, Kalman filters have been employed to estimate vehicle density and space mean speeds on highways [22,23]. These studies demonstrated that Kalman filters could provide reliable state estimates, but their primary focus was on highway traffic, where traffic dynamics are more predictable compared to urban traffic networks. As a result, their applicability to signalized intersections remains limited.

Antoniou et al. [24] applied a nonlinear Kalman filter algorithm to calibrate online dynamic traffic assignment models. The proposed method achieved high accuracy and computational efficiency. However, the study mainly focused on large-scale traffic networks and did not address traffic state estimation at the microscopic level, such as signalized intersections, which require more granular and dynamic estimation methods.

Emami et al. [25] utilized a Kalman filter algorithm for short-term traffic flow prediction in a connected vehicle (CV) environment, while the study demonstrated the effectiveness of incorporating CV data into prediction models, it assumes a high market penetration rate of connected vehicles. This assumption may not hold in real-world scenarios with lower CV adoption rates, limiting the method’s practical applicability.

Bekiaris-Liberis et al. [26] used a Kalman filter to estimate mixed traffic density on highways using the average speed of connected vehicles. This method showed high estimation accuracy but relied heavily on connected vehicle data, making it less effective for low CV penetration levels. Additionally, the study focused on highway traffic conditions, where traffic flow is more homogeneous compared to urban settings.

Kalman filters have also been applied to turning movement estimation at signalized intersections such as the work of Jiao et al. [27], which proposed a Bayesian approach combining a back-propagation neural network and a revised Kalman filter. This method achieved high estimation accuracy by leveraging both historical and real-time data. However, the reliance on historical data can be a significant limitation in situations where such data are scarce or unavailable, and the computational complexity of the method may hinder real-time applications.

Zhang et al. [28] employed a Kalman filter technique based on an extended cell transmission model (ECTM) to estimate TMs for adaptive traffic signal control systems, while the method provided reliable traffic state estimates, its accuracy decreased during highly dynamic traffic conditions, such as sudden fluctuations in demand, which are common at signalized intersections.

Enjedani et al. [29] developed a Kalman filter TM estimator using probe vehicle data. The study showed improved estimation accuracy by considering probe data representative of total traffic flow at low CV penetration levels. However, the method’s reliance on historical data limits its applicability to short-term TM estimation and real-time traffic signal optimization.

Hu et al. [30] applied an Extended Kalman Filter (EKF) for queue estimation at signalized intersections, integrating a machine learning-based shockwave queue propagation model. The study demonstrated accurate real-time queue estimates. However, the EKF’s accuracy is highly dependent on the quality of the machine learning model training, which can be challenging due to the extensive historical data requirement.

#### Adaptive Kalman Filtering

Previous Kalman filtering applications are based on the prior knowledge of the error parameters in the filter, which are the measurement noise covariance matrix R, as well as the process noise covariance matrix Q. Most of the previous work utilized historical data to estimate fixed error matrices, assuming that the error ranges will remain constant. This assumption might not always be true, in addition, historical data may not be available in many applications. As such, the concept of adaptive Kalman filtering emerged, where prior knowledge of the noise matrices Q and R are not required.

An adaptive Kalman filtering method was first proposed by Mehra in 1970 [31], where the author developed a technique to obtain unbiased estimates and consistent estimates of R and Q for discrete systems. This technique is considered useful in the cases where the noise matrices are unknown. The proposed technique is also extended to continuous filtering systems, with high filtering efficiency. Brown and Rutan, 1985 [32] also proposed a method to obtain the unknown measurement error matrix R from an empirically chosen window. The study showed significant accuracy improvement in the estimated parameters. Another study by Karasalo and Hu, 2011 [33] also proposed a procedure to estimate the noise matrix Q. The study used system models $\dot{x}=f\left(s\right),y=h\left(x\right)+v$ to recover the observation h(x) without prior knowledge of the system dynamics.

Adaptive filtering has also been used for many applications in vehicle tracking and traffic state estimation. For example, Hu et al., 2003 applied an adaptive Kalman filter algorithm to GPS data for vehicle navigation [34]. The study showed the efficiency of the adaptive algorithm in capturing sudden changes in the vehicle’s motion as well as accounting for measurement errors. Some studies have explored adaptive filtering approaches for traffic state estimation on highways. For instance, Chen et al. [35] used an adaptive rolling smoothing (ARS) method to estimate the traffic flow rate, speed, and time occupancy to mitigate traffic congestion dynamics. The ARS method adjusts filter parameters in a rolling horizon, making it suitable for online applications by dynamically estimating measurement noise from real-time data. However, this method is primarily designed for highways and does not account for the unique challenges of traffic state estimation at signalized intersections.

Similarly, Wang et al. [36,37] employed adaptive extended Kalman filters to estimate traffic states on highways, while these methods effectively addressed traffic congestion dynamics on freeway segments, they overlooked traffic state estimation at urban intersections, where traffic flow patterns are more complex due to the presence of traffic signals, queuing dynamics, and turning movements.

In conclusion, the use of Kalman filtering has been extensively studied in traffic state, turning movement, density, and queue estimation. However, most existing studies focus on overall traffic state estimation without differentiating by turning movement, which is critical for adaptive traffic signal controllers that rely on movement-specific data. Additionally, the majority of traffic state estimation methods have been applied on highways, where traffic flow is more predictable, while fewer efforts address the more complex and dynamic conditions at intersection approaches. Many methods incorporate historical traffic data within Kalman filters to account for estimation errors, but these data are often unavailable or unreliable in real-world applications. The reliance on machine learning models alongside Kalman filters further limits practical applicability, as these methods depend heavily on the availability of training data and cannot generalize beyond the scope of the historical data used. Furthermore, some studies assume high connected vehicle (CV) penetration rates, which is unrealistic in many traffic systems and introduces additional computational complexity that may hinder real-time implementation. Finally, most existing approaches fail to provide real-time updates at short intervals, making them less effective for adaptive traffic signal control in rapidly changing traffic conditions. Addressing these shortcomings is crucial to developing more accurate, movement-specific, and practical traffic state estimation methods for real-time traffic management at intersections.

### 2.4. Research Gap and Study Contribution

This study contributes to the existing body of knowledge by proposing a two-stage Kalman filtering approach for traffic state estimation and prediction in the vicinity of traffic signals. The proposed approach bridges the research gap in the literature in the following ways:

1. The proposed system offers real-time traffic state estimations and predictions for short-term variable horizons, addressing a gap in the current state-of-the-art system.
2. Unlike many studies that focus on traffic state estimation for highways, this system is specifically designed to estimate and predict traffic states in the vicinity of intersections, making it highly relevant for urban traffic signal control.
3. The system eliminates the need for historical traffic data by directly estimating the Kalman filter error covariance matrices using real-time data, enhancing practicality compared to machine learning models that require extensive historical training data.
4. It simultaneously integrates the real-time estimation and prediction of turning movements (TMs), traffic stream density, and queue size—a unique feature that, to the best of the authors’ knowledge, has not been addressed by any existing study.
5. Unlike state-of-the-art approaches, the proposed approach is computationally efficient, making it well-suited for advanced real-time traffic signal controllers compared to other methods that require significant computational resources or model training.

## 3. Design of Adaptive Kalman Filter

### 3.1. System Overview

A Kalman filter is an efficient recursive minimum variance estimator that follows a prediction–correction scheme. The Kalman filter integrates the recent previous state estimate with the current state observation to perform state estimation and future state projections. This algorithm was first developed by Kalman in 1960 [38]. The filter uses previous state estimates, new state observations, and projections of previous states to generate refined estimates of the current state variables. This is done by separating the random noise inherent in the observations and fusion with projections of previous states. Kalman filters are used in numerous industrial applications such as object tracking, signal processing, and navigation [39], which makes it suitable for time series applications to estimate TMs on a real-time basis.

In this study, a Kalman filter algorithm was employed in a two-stage approach to estimate and predict TMs in the first stage and density and queue size in the second stage.

The Kalman filter algorithm is utilized within a two-stage approach. At the first stage, it is utilized for TM estimation, while at the second stage, it is utilized for density and queue size estimation.

**Figure 1:**
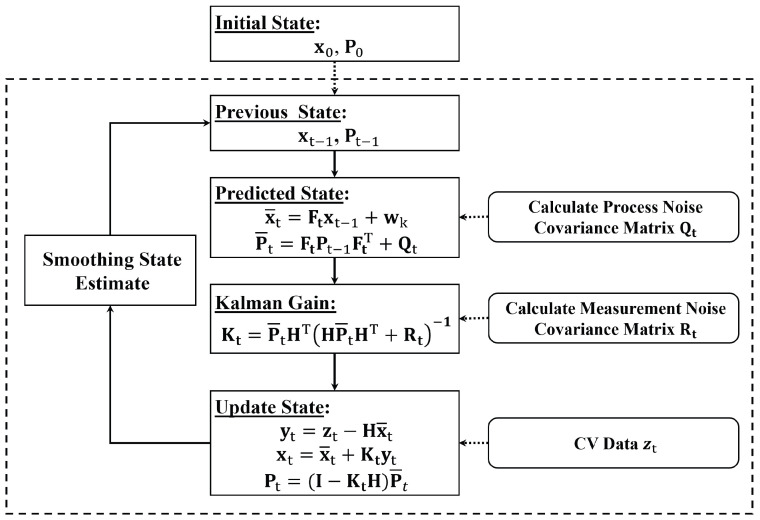
Kalman filter process.

As depicted in Figure 1, the algorithm follows a multi-stage process for estimation and prediction, where the system makes state predictions in one stage and then the state is updated using observed data in another stage. In this system, historical data are not required to calibrate the error matrices, but the current observations and the perceived market penetration rate are used to quantify the process and observation noise matrices.

**Figure 2:**
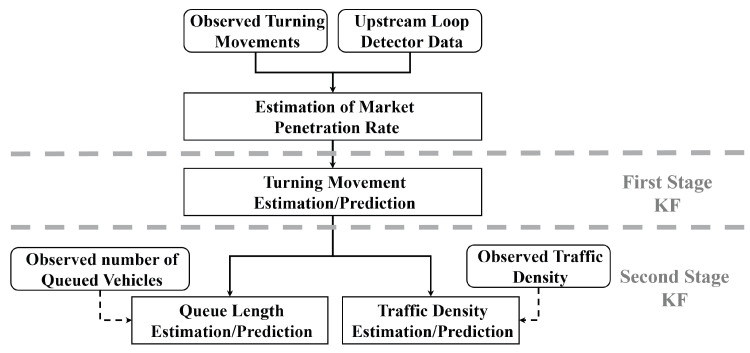
General process overview.

Figure 2 shows the general estimation and prediction framework that utilizes the observed measurements from probe vehicles and stationary detectors to estimate the traffic state parameters. First, the market penetration rate is estimated in real-time. Then, the first-stage Kalman filter estimates and predicts the turning movement rates for the current and the next time steps, respectively. Second, the predicted turning rates are used to estimate and predict the queue length and the traffic density in the second stage of the Kalman filtering process.

As shown in Figure 1, the Kalman filter uses the previous traffic state estimates to obtain the predicted state using the process matrix $F_{t}$. The prediction function incorporates the time-varying process noise covariance matrix $Q_{t}$. The process matrix $F_{t}$ represents the rate of change in loop detector readings, calculated using current and previous loop detector data. This rate is assumed constant for a single prediction step, which is reasonable for short prediction periods of 10–30 s.

The Kalman gain $K_{t}$ is calculated using the updated state covariance matrix ${\bar{P}}_{t}$ and the time-varying measurement noise covariance matrix $R_{t}$. In the update step, the estimated state variable and covariance matrix are calculated upon receiving probe vehicle observations. Finally, exponential smoothing is applied to enhance the filter’s performance. Subsequent sections provide more elaboration on these steps.

### 3.2. State Variables

In this study, because the objective is to estimate TMs at intersection approaches in the first stage, the state variables are defined by the traffic flow rate (in veh/h) turning right ($x_{rt}$), heading through ($x_{thr}$), and turning left ($x_{lt}$), respectively. The state vector x(n×1), where *n* is the number of state variables, is defined as follows:

$$x={\left[\begin{array}{ccc} x_{rt} & x_{thr} & x_{lt} \end{array}\right]}^{T}$$

These TM state variables can be observed from the combination of probe vehicle trajectory data and stationary detector data, where the probe CV vehicles broadcast TMs, which represent samples of the total flows. The Kalman filter approach employs a rollback method to determine probe vehicle headings. This means the system goes back in time until it locates at least one vehicle for each TM. If no probe vehicles are observed turning in a particular direction, the system considers TM as zero. These observations are then balanced by the total approaching traffic flow obtained from the upstream loop detector. The accuracy of the TM observations relies on the current level of market penetration at each experiment, while the detectors capture the approaching volumes.

In the second stage, a single variate Kalman filter is applied for each direction to estimate and predict the traffic density(in veh/km) on the upstream link as well as the number of queued vehicles per movement (in vehicles for the left and through movements). Upstream locations and speeds of probe vehicles are used as measurements for the estimation of the current upstream density and queues.

### 3.3. Prediction Step

#### 3.3.1. State Transition Function

In this step, the current state vector is projected to the short-term future as a process model. The Kalman filters future projections of one step ahead in the future, typically referred to as a prediction using the current and past state observations.

The state transition function, as shown in Equation (1), utilizes the process matrix $F_{t}$, the current state x, and the state transition noise $w_{k}$ to provide the next state vector ${\bar{x}}_{t}\;\left(n\times 1\right)$, which represents the predicted state vector of the next time step. It is noted that the state transition function $F_{t}$ is time-varying based on the current and previous loop detector observations. The transition function is shown as follows:

(1) $${\bar{x}}_{t}=F_{t}x_{t}+w_{k}$$

where $w_{k};\left(n\times 1\right)∼N\left(0,Q_{t}\right)$ denotes the state transition (process) noise with dimensions n×1, following a normal distribution with mean 0 and process noise matrix $Q_{t}$.

The state covariance matrix ${\bar{P}}_{t}$ is calculated in this step using the current covariance matrix $P_{t}$, the process matrix $F_{t}$, and the process noise covariance matrix $Q_{t}$. The state covariance update is represented in Equation (2) as follows:

(2) $${\bar{P}}_{t}=F_{t}P_{t}F_{t}^{T}+Q_{t}$$

The process noise matrix $Q_{t}$ will be explored in the next subsection.

#### 3.3.2. Process Noise Covariance Matrix

In the context of this problem, the time-varying process noise covariance matrix $Q_{t};\left(n\times n\right)$ is defined as the matrix composed of the prediction error variance of each of the state variables. $Q_{t}$ is recalculated at each iteration in each of the filter stages to account for the prediction error.

The process noise in the first stage Kalman filter represents the prediction errors caused by the assumption that the growth factor is identical for all turning movements. Since the actual growth factor will not be the same for all turning movements, the error should be quantified using the observed growth factor for each individual turning movement obtained from CVs, as well as the market penetration level. As such, the standard error formula (Equation (3)) quantifies how the observed individual growth rates vary from the actual individual growth rates for the population. Subsequently, the matrix $Q_{t}$ is comprised of the standard error for each turning movement on the matrix diagonal.

(3) $$\mathrm{Standard}\mathrm{Error}\left(S.E.\right)=Z\sqrt{\sigma_{p}^{2}/n}=Z\sqrt{p\times (1-p)/n}$$

where $\sigma_{p}^{2}$ corresponds to p×(1−p) according to the Bernoulli distribution. *Z* is the Z-score of the selected confidence level; *p* is the proportion of the TM from the total approach flow; and *n* is the sample size, which is the number of observed probe vehicles in a certain time window. The iterative computation of the process noise matrix relies on the continuous broadcast of probe vehicle data at each time step. This involves collecting observations within a rolling-back horizon and ensuring that the rollback period includes at least one probe vehicle for each of the TMs, which is essential to ensure a reliable and representative sample.

In the second stage, since the density and queue size predictions are based on the estimated TMs in the first stage, the covariance matrix $P_{t}$, which represents the confidence in TM estimates, is used to calculate the prediction covariance matrix, resulting in an adaptive Kalman filter.

#### 3.3.3. The Process Model: Stage 1

The process model $F_{t}$ is derived at each Kalman filtering stage based on the dynamic nature of the state variable. The process model for the first stage filter is provided in Equation (4), where the process matrix $F_{t}$ is a diagonal matrix comprised of the growth factor $GF_{t}$ over short intervals using the traffic flow continuity (conservation of the number of vehicles) Equation (5). In this stage, the process model $F_{t}$ is based on the growth factors ($GF_{t}$) estimated using loop detector measurements, as shown in Equation (6). The most recent observed flow growth rate is assumed to remain constant, which is a reasonable assumption over small time intervals.

(4) $$F_{t}=diag\left(GF_{t},GF_{t},GF_{t}\right)$$

(5) $$\frac{\partial k(x,t)}{\partial t}+\frac{\partial q(x,t)}{\partial x}=0$$

where *x* denotes the spatial coordinate in the traffic flow direction, *k* is the traffic stream density, *q* is the traffic flow, and *t* is the time instant.

(6) $$GF_{t}=L_{t}/L_{t-∆t}$$

where $L_{t}$ represents the traffic flow measured upstream via loop detectors or other stationary sensors.

#### 3.3.4. The Process Model: Stage 2

In the second stage, the input-output queue estimation method [15] is used to predict the number of queued vehicles as well as the traffic density. The turning movements traffic flow, obtained from the first-stage Kalman filter, are used to determine the arrival flows. The traffic signal indication for each movement on each approach is also used to determine the departure rate. The traffic density process model employed a method similar to the queue estimation process model. The equation below utilizes the input–output model to calculate the predicted vehicle queue (or the traffic density).

(7) $$u_{t+∆t}=\left\{\begin{array}{cc} u_{t}+q_{\mathrm{in}}\times ∆t-q_{\mathrm{out}}\times \left(∆t-t_{\mathrm{lost}}\right), & \mathrm{for}\;\mathrm{green}\;\mathrm{phases}.\\ u_{t}+q_{\mathrm{in}}\times ∆t-q_{\mathrm{out}}\times t_{\mathrm{eg}}, & \mathrm{for}\;\mathrm{red}\;\mathrm{phases}. \end{array}\right.$$

where $u_{t}$ is the current vehicle queue or the traffic density, $q_{in}$ is the average traffic flow obtained from the first stage KF, and $q_{out}$ is the average discharge rate.

### 3.4. Update Step

#### 3.4.1. Measurement Function

The Kalman filter refines the projected state using probe vehicle observations, represented by the measurement vector $z_{t}$. As shown in Equation (8), the residual vector $y_{t}$ is calculated using the predicted state vector ${\bar{x}}_{t}$ and the measurement vector $z_{t}$. Because the Kalman filter computes the residual vector in the measurement space, matrix H is used for the transition. In the second stage Kalman filter, probe vehicles are used to measure the number of stopped vehicles in the queue. It is noted that the matrix H is omitted in the second stage Kalman filter because the density and queue size estimation problem is univariate, where the matrix H equals [1].

(8) $$y_{t}=z_{t}-H{\bar{x}}_{t}$$

where $z_{t}$ is the measurement vector defined as ${\left[\begin{array}{ccc} x_{thr} & x_{rt} & x_{lt} \end{array}\right]}^{T}$ in the first stage Kalman filter at time *t*, and H;(m×n) is the state-to-measurement transition matrix, defined as follows:

$$H=\left[\begin{array}{ccc} 1 & 0 & 0\\ 0 & 1 & 0\\ 0 & 0 & 1\\ 1 & 1 & 1 \end{array}\right]$$

#### 3.4.2. Measurement Noise Matrix

The Kalman filter employs the measurement noise covariance matrix $R_{t};\left(n\times n\right)$ to capture the inherent uncertainty in the measurement vector. This matrix quantifies the variance of expected errors in these measurements, which is based on the level of market penetration of the probe vehicle data. The calculation procedure of the $R_{t}$ matrix is based on the sampling error approach, where the matrix is calculated at each KF update iteration using the measured number of vehicles and the detector data representing the total number of vehicles.

The error variance is derived from the sampling error computed using Equation (3), which quantifies how much confidence we can place on the observed number of TMs. Subsequently, the Kalman gain $K_{t}$ is calculated using Equation (9) and used to update the state estimate x and the state covariance matrix $P_{t}$, as shown in Equations (10) and (11). The variable x is considered a *recursive* solution of the optimal state estimation problem, which means that the system state can be estimated solely based on the previous state estimate and the new observation $y_{t}$. This property of the approach is computationally efficient and makes it suitable for real-time applications [22].

(9) $$K_{t}={\bar{P}}_{t}H^{T}{\left(H{\bar{P}}_{t}H^{T}+R_{t}\right)}^{-1}$$

(10) $$x_{t}={\bar{x}}_{t}+K_{t}y_{t}$$

(11) $$P_{t}=\left(I-K_{t}H\right){\bar{P}}_{t}$$

#### 3.4.3. The Observation Model: Stage 1

The observation model $z_{t}$ is derived at each Kalman filtering stage based on the dynamic nature of the state variable. In the first stage, The observation model is given in Equation (12), where the turning movement measurement represents the flow rate of probe vehicles within a dynamic rollback window, factored by the market penetration rate. This window is selected dynamically so that at least one connected vehicle is observed at each turning movement, assuming that probe vehicles are present at all turning movements.

(12) $$z_{t}=\frac{{\left[N_{\mathrm{thr},t},N_{\mathrm{rt},t},N_{\mathrm{lt},t},N_{\mathrm{tot},\;t}\right]}^{T}}{MP_{t}}$$

where $N_{d}$ represents the flow rate of probe vehicles for direction *d*. $MP_{t}$ represents the market penetration proportion at time *t*, as calculated in Section 4.4.

#### 3.4.4. The Observation Model: Stage 2

In the second stage, the positions and speeds of the probe vehicles are used to infer the number of queued vehicles, and the traffic density within the measurement region. The inferred market penetration rate is used to estimate the total number of vehicles. Equation (13) below shows the observation model for the second stage; there, the measurement vector depends on the vehicle longitudinal position on the link (*x*) at each turning movement at time *t*, as well as the market penetration rate.

(13) $$z_{t}=g\left[x_{v,dir,t},MP_{t}\right]$$

### 3.5. Estimate Smoothing

The probe vehicle observations are subject to sudden changes or outliers, which can significantly impact the performance of the Kalman filter’s recursive process. As such, an exponential smoothing technique is applied to the Kalman-filtered state estimates to further reduce the noise and enhance their accuracy and precision. Equation (14) shows the exponential smoothing formula, in which the value α variables are tuned according to the level of market penetration. In addition, ${\tilde{x}}_{t}$ and $\tilde{x}\left(t-∆t\right)$ represents the smoothed state variables at time *t* and (t−∆t), respectively.

The α values close to zero put more weight on past values, making the forecast smoother and slower to react to recent changes, which is used to limit fluctuations in the traffic state estimates due to the high errors at low market penetration levels, while higher values close to 1.0 put more weight to observations at higher confidence cases. In this study, α values vary linearly from 0.4 to 1.0 linearly based on the value of market penetration level, where the value 0.4 corresponds to the minimum MPL and 1.0 corresponds to MPL = 100. The minimum value of α (0.4) is calculated using the trial-and-error method by testing different values of α and selecting the one that minimizes the forecast error.

(14) $${\tilde{x}}_{t}=\alpha x_{t}+\left(1-\alpha \right){\tilde{x}}_{t-∆t}$$

## 4. Experimental Design

### 4.1. Data Collection

**Figure 3:**
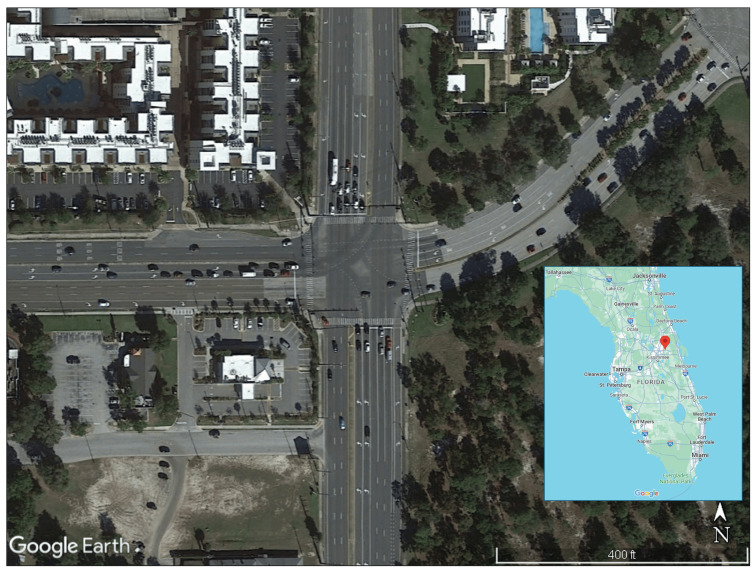
Four-legged signalized intersection at Alafaya Trail and University Boulevard in Orlando, FL, USA.

The evaluation of the proposed system involved a comprehensive analysis of real-world trajectory data sourced from a drone-based dataset. The detailed contextual information about the studied intersections is provided in the original publication by Zheng et al., 2023 [40]. The dataset, which was specifically designed for urban traffic studies, focuses on the traffic dynamics at a four-legged signalized intersection situated at the intersection of Alafaya Trail and University Boulevard in Orlando, Florida. This location, illustrated in Figure 3, serves as a case study for assessing the effectiveness of the two-stage approach for TM and queue size estimations. The choice of this dataset is particularly significant, as it provides a realistic and dynamic representation of urban traffic scenarios, offering insights into the algorithm’s performance under real-world conditions of actual traffic patterns and oscillations. In addition, using this dataset ensures that the evaluation process is performed in applicable scenarios, enhancing the credibility of the proposed system’s performance assessment.

### 4.2. Upstream Detector and CV Data

The raw trajectory data are transformed to mimic real-time probe vehicle data and upstream loop detector information, where traffic flow features such as the traffic flow and individual vehicles’ trajectories are extracted. The total flow count on each approach is computed using the raw trajectory data. In addition, based on the market penetration level in each experiment, randomly sampled trajectories are selected to represent probe vehicles, where they broadcast their headings upon entering the intersection zone. The real-time information, including detector data and probe vehicle data, is fed to the two-stage approach. The evaluation utilized one hour of trajectory data collected at a frequency of 30 frames per second. Table 1 shows the total hourly demand for each approach per direction according to the data.

### 4.3. Intersection Microscopic Simulation

#### 4.3.1. INTEGRATION Background

INTEGRATION is a microscopic traffic assignment and simulation software developed and maintained at the Center for Sustainable Mobility (CSM) [41,42,43,44,45,46]. INTEGRATION was first established in the late 1980s, with developments and enhancements continuing through today. It was conceived as an integrated simulation and traffic assignment model that performs traffic simulations by tracking the movement of individual vehicles every 1/10th of a second. This allows the detailed analysis of lane-changing and longitudinal vehicle movements and their resulting shock wave propagations. It also permits considerable flexibility in representing spatial and temporal variations in traffic conditions. The INTEGRATION software v.2.4 incorporates a point mass vehicle dynamics model that computes the vehicle’s tractive effort, aerodynamic, rolling, and grade-resistance forces. The vehicle dynamics model is fully integrated in the car-following model [47,48,49,50]. In addition to estimating stops and delays [51,52], the model can also estimate the crash risk [53], the fuel consumed by vehicles [44,54] and the vehicular emissions. The INTEGRATION model has not only been validated against standard traffic flow theory [51,52] but has also been used to evaluate large-scale real-life applications [55,56].

#### 4.3.2. Intersection Modeling

**Figure 4:**
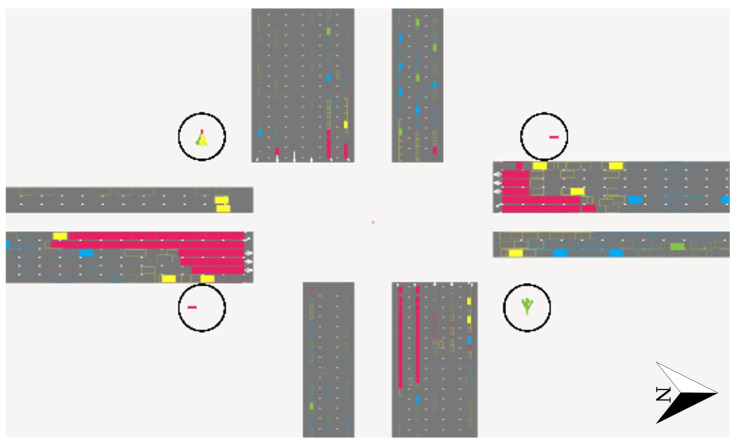
Snapshot of INTEGRATION microscopic simulation model.

A microscopic simulation model was developed using the INTEGRATION microscopic simulation software for the same four-legged intersection to test the algorithm for conditions not observed in the field. The vehicle arrivals were modeled to match the observed vehicle counts in the raw vehicle trajectory data for discretized intervals of 10 s for the entire hour. The field settings, such as the speed limits, traffic flow rate, and the geometric characteristics of the intersection, are replicated in the model. Figure 4 shows a snapshot of the developed micro-simulation model.

This model was used to evaluate the developed two-stage KF approach, in which proper signal timings are proposed, and the traffic state estimates are made based on the modeled signal timings. This approach mitigates data collection errors caused by time mismatches between vehicle trajectories and signal timings. The proposed two-stage Kalman filter approach was applied to the model using upstream loop detectors and probe vehicles at various levels of market penetration ranging from 5% to 100%. In addition, actual queue sizes and densities were observed in the model and compared with the estimated values for the simulation period.

### 4.4. Estimation of the Market Penetration Level

The underlying total market penetration (MP) level is required by the Kalman filter algorithm, yet it is unknown to the system. As such, the MP rate for the total traffic is estimated in real-time using a basic process that utilizes probe vehicle data as well as loop detector data. Within each temporal window, the number of in-flow probe vehicles is compared with the total traffic flow that enters the link. As such, the perceived MP level for the total flow is estimated for each link. It is noted that the market penetration level may differ for each turning movement, and since the total number of vehicles per turning movement is unknown, the total MP level is used and the sampling error is taken into account in the measurement noise matrix.

## 5. Results and Discussion

### 5.1. Evaluation Baseline

The proposed Kalman filter method is evaluated by comparing its results with the ground truth derived from trajectory data. The performance metrics of the KF method are also analyzed and benchmarked against connected vehicle observations. This comparison takes into account varying market penetration levels observed in different simulation experiments to provide a comprehensive understanding of the KF method’s effectiveness in real-world applications.

### 5.2. TM Estimation and Prediction Results

**Figure 5:**
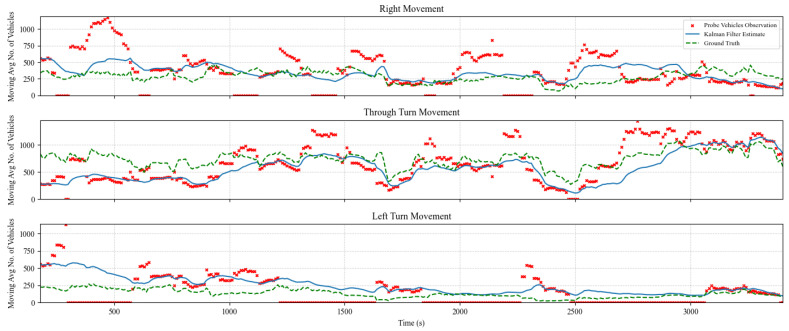
Comparison between Kalman filter-derived TM counts versus CV observations and ground truth counts for the EB approach at a MPL of 5%.

**Figure 6:**
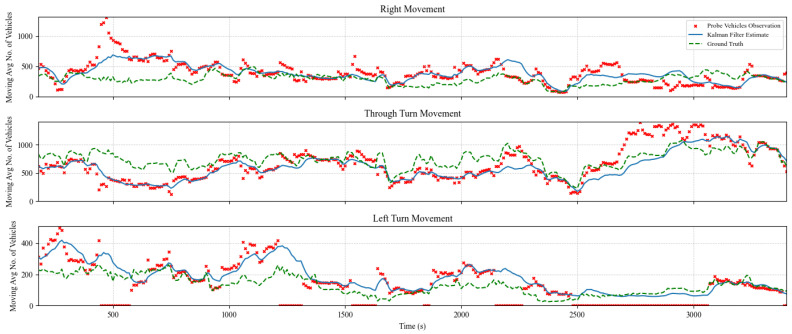
Comparison between Kalman filter-derived TM counts versus CV observations and ground truth counts for the EB approach at market penetration = 10%.

**Figure 7:**
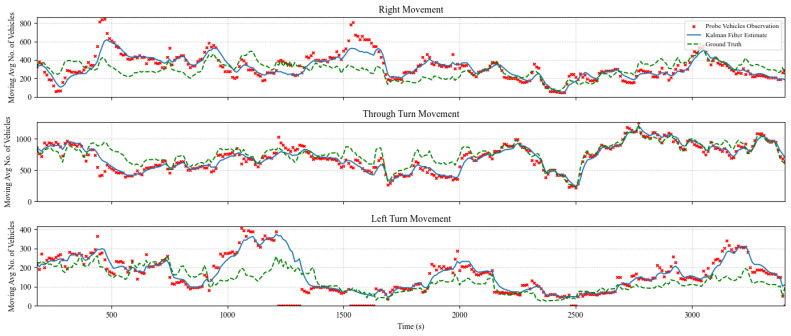
Comparison between Kalman filter-derived TM counts versus CV observations and ground truth counts for the EB approach at market penetration = 20%.

The TM estimation algorithm was evaluated for multiple cases with prediction horizons of 1, 5, and 10 s. In addition, several experiments were performed considering various levels of market penetration of probe vehicles ranging from 5% to 100%. Figure 5, Figure 6 and Figure 7 show the algorithm performance in estimating TMs on the east-bound (EB) intersection approach at a market penetration level of 5%, 10%, and 20%, respectively. Each figure shows the hourly moving average number of turning vehicles for one hour for each of the following three TMs: through, right, and left. The plots show the distribution of actual TMs compared to the estimated number of TM counts as well as the probe vehicles. Figure 5, Figure 6 and Figure 7 illustrate the TM estimation error compared to the filtered estimates, highlighting the efficiency of the proposed Kalman filter algorithm, where the estimated TMs have significantly less noise than those estimated by CV data, even at low levels of market penetration.

#### 5.2.1. Accuracy of Estimation and Prediction

To further assess the estimation errors of the proposed algorithm, Table 2 provides a comparison of the standard deviations (SD) associated with TM estimates derived from both the probe data and Kalman filtering approaches for market penetration levels of 5%, 10%, and 20%. Notably, the Kalman filtering approach demonstrates a significant enhancement in the SD of current real-time TM estimates. For instance, at a market penetration level of 5%, the proposed Kalman filter approach reduced the SD by 48%. Additionally, the SD of the Kalman filtering predictions is 92.8 vehicles/h, further showcasing the accuracy of the Kalman filter in predicting TMs even at low levels of market penetration.

**Figure 8:**
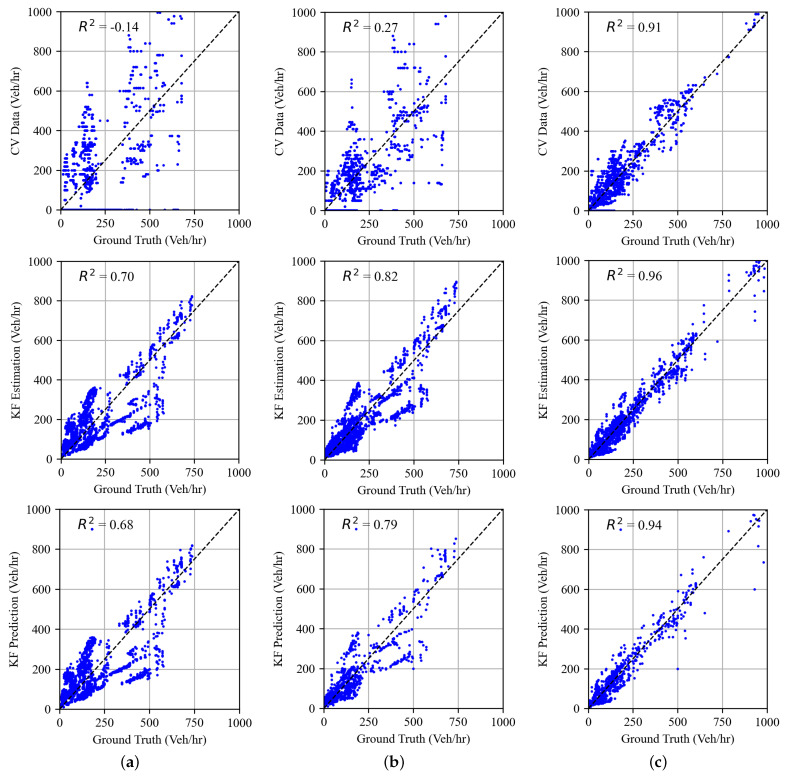
Correspondence between TM estimates and ground truth. (**a**) Market penetration = 5%; (**b**) market penetration = 10%; (**c**) market penetration = 20%.

The findings were also evaluated by the results of the analysis depicted in Figure 8. For instance, at the 5% penetration level, the coefficient of determination $\left(R^{2}\right)$ advanced from −0.14 for probe-vehicle-estimated TMs to a significantly improved 0.70 for Kalman filter-estimated TMs at a market penetration level of 5%. Moreover, the Kalman filter predictions exhibit a coefficient of determination of 0.68, which is a substantial enhancement over the accuracy achieved when using only probe vehicle data. These results illustrate the effectiveness of the Kalman filter approach in refining TM estimates and providing reliable TM estimates that are representative of real-time traffic dynamics.

**Figure 9:**
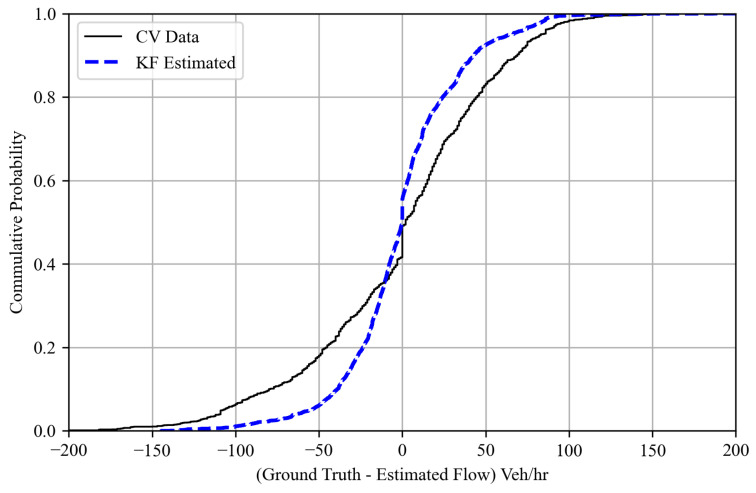
The cumulative probability of the difference between the ground truth and Kalman filter-derived estimated flows.

**Figure 10:**
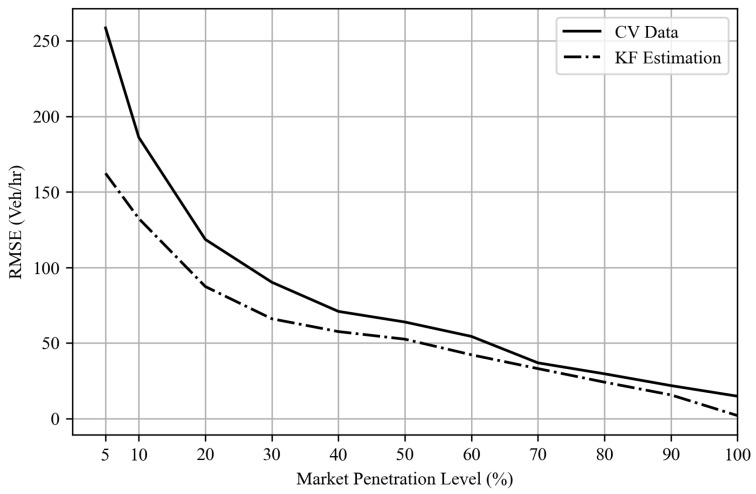
TM estimation accuracy results for different levels of market penetration.

**Figure 11:**
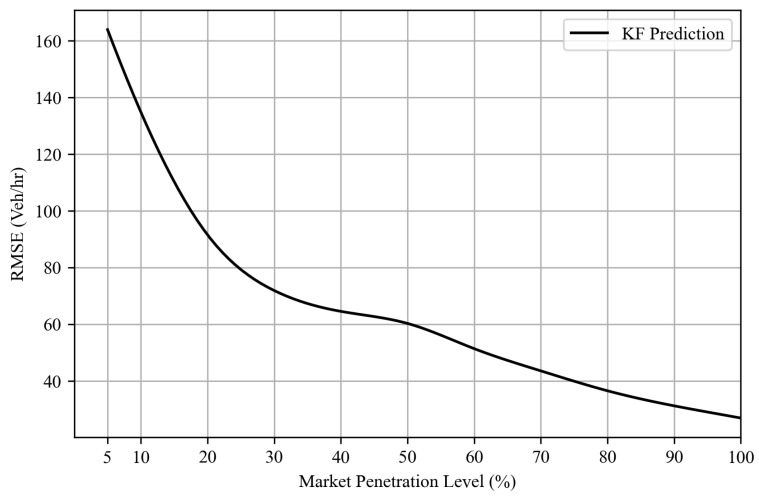
TM prediction RMSE results for different levels of market penetration.

Figure 9 shows the probability distribution of the difference between the ground truth approach flows and the corresponding estimates/predictions generated by the Kalman filter algorithm. Notably, the deviations observed in TM estimates derived from probe vehicle data are significantly reduced. Furthermore, Figure 10 shows the root-mean-squared error (RMSE) values across various levels of CV market penetration implemented in this study. The outcomes reveal that the Kalman filter algorithm consistently outperforms solely CV-derived estimates in capturing the current system state. Furthermore, even at low CV market penetration levels, the Kalman filter algorithm demonstrates improved predictive capabilities for TM counts, with an RMSE value ranging from 164 to 27 vehicles/h at various MP levels, as shown in Figure 11, where the level of error is significantly lower than the estimated TM by probe vehicle data alone. This underlines the reliability of the proposed Kalman filter approach, which qualifies this algorithm to be used as a tool to provide TM counts for queue size estimation algorithms, as performed in this research. The algorithm also qualifies to be integrated with real-time traffic analysis and signal control systems, with varying degrees of CV market penetration levels.

#### 5.2.2. Validation of INTEGRATION Micro-Simulation Model

**Figure 12:**
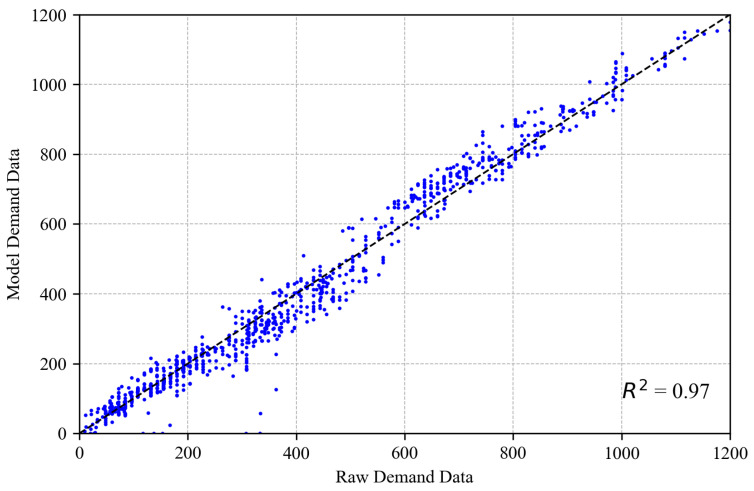
Demand validation.

**Figure 13:**
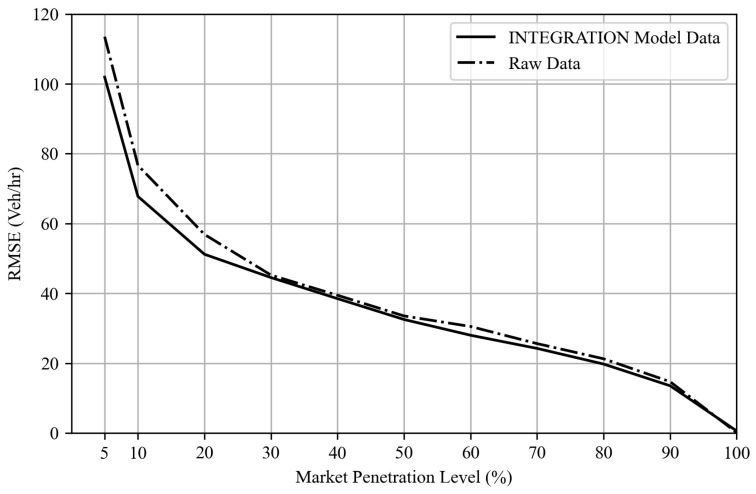
Validation of the developed INTEGRATION microscopic model.

To validate the input demand, Figure 12 shows that the simulated loop detector data match the observed demands derived from the raw trajectory data ($R^{2}=0.97$). Furthermore, to validate the use of this model for further queuing analysis, the Kalman filter TM estimation approach was applied to both the simulated outputs and the raw trajectory data. Figure 13 shows a comparison between the RMSE of both estimates using raw trajectory data versus the INTEGRATION model output trajectory dataset. Figure 13 shows high consistency between both outputs. The minor difference is due to the raw data’s higher resolution of vehicle trajectory data (30 frames per second versus the simulated data of 1 frame per second), which translates into more randomness and a higher estimation error, as shown in Figure 13. These results enable us to confidently evaluate the proposed queue estimation scheme using the developed microscopic simulation model data.

### 5.3. Queue Size and Density Estimation Results

In this subsection, the second stage of the proposed approach is evaluated. The Kalman filter method was used for queue size and density estimation and compared with the baseline estimates using solely probe vehicle data. The TM estimates conducted at the first stage were utilized to estimate and predict the real-time queue size and density. The two-stage approach was implemented for the isolated signalized intersection for multiple levels of probe vehicle penetration ranging from 5% to 100% for each prediction horizon of 5 and 10 s.

**Figure 14:**
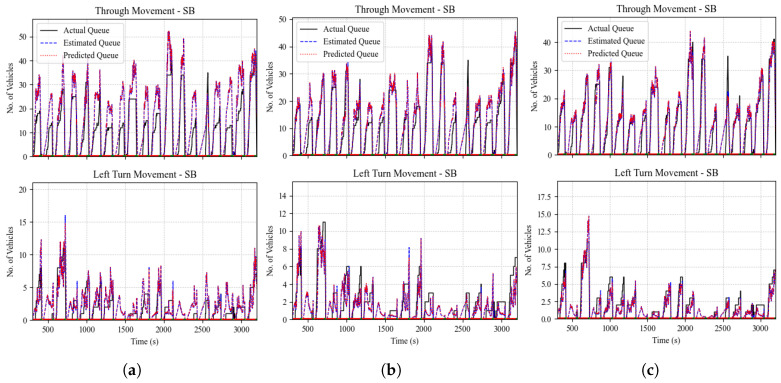
Comparison between queue estimation using the Kalman filter method and ground truth. (**a**) Market penetration = 5%; (**b**) market penetration = 10%; (**c**) market penetration = 20%.

**Figure 15:**
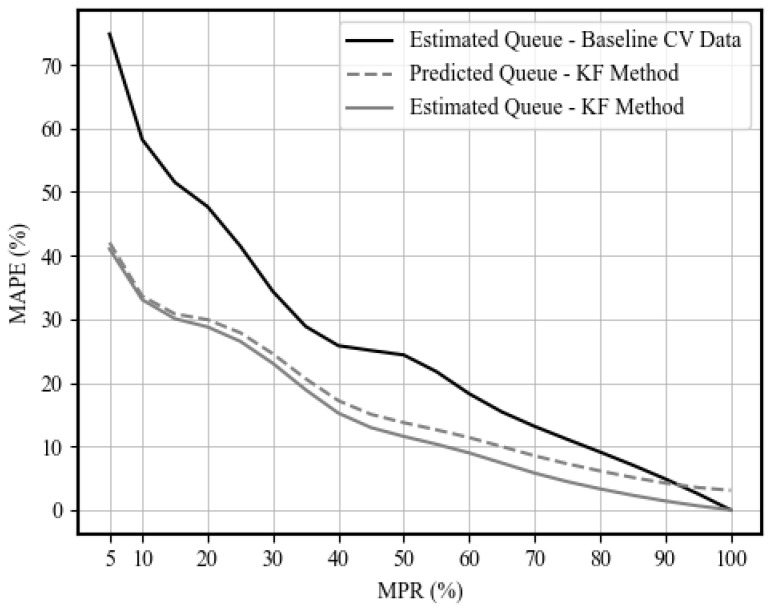
Queue estimation and prediction MOP results at different levels of market penetration.

Figure 14 shows the queue size estimation results for the through and left TMs at the north-bound (NB) approach of the intersection for market penetration levels of 5%, 10%, and 20%, respectively. Figure 14 shows the performance of the queue estimation algorithm using Kalman filters, where it captures the general queuing pattern, even at low levels of market penetration. The estimation accuracy is further quantified in Figure 15, which shows the performance of the proposed estimation scheme at various levels of market penetration ranging from 5% to 100%. The mean absolute percentage error (MAPE) and the root mean square error (RMSE) are the measures of performance (MOP) chosen for evaluation.

Figure 15 illustrates the performance evaluation results of the queue estimation and prediction. Figure 15 shows that the use of Kalman filtering for queue size estimation outperforms the baseline estimates using the probe vehicle data by up to 32.8%, which highlights the filter’s capability to reduce the estimation noise at low levels of market penetration. However, at high levels of market penetration, the results show an insignificant impact from using Kalman filters compared to the sole use of probe vehicle data to derive queue size estimates. Figure 15 also shows the prediction quantification results, where the level of error is consistent with the state estimation error results across various MP levels.

**Figure 16:**
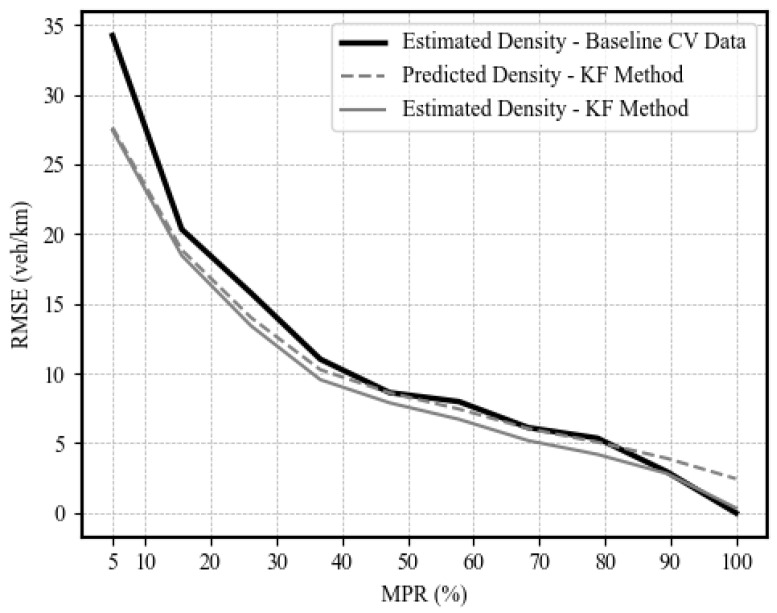
Density estimation and prediction RMSE results at different levels of market penetration.

To quantify the estimation of upstream traffic density, Figure 16 shows the RMSE of the traffic density estimates (the density of vehicles traveling at upstream links in veh/km) and predictions using Kalman filters compared to the baseline method. Figure 16 shows improved estimation at various market penetration levels, where the RMSE improved by up to 21%. Figure 16 also shows the prediction performance of the filter, whose prediction accuracy is consistent with the estimation accuracy.

### 5.4. Discussion

The previous subsections demonstrated the efficiency of the proposed two-stage approach for TM, density, and queue size estimation and prediction using Kalman filters. The proposed system showed enhanced state estimation and prediction and its applicability for field implementation and integration with traffic signal control systems. Specifically, the proposed two-stage approach improved traffic state estimates and predictions demonstrating significant system performance at low levels of market penetration. However, the results showed a comparatively marginal enhancement achieved by the proposed approach at higher penetration levels compared to the baseline. This is attributed to the increased accuracy and reduced noise inherent in the probe vehicle data at higher penetration levels, diminishing the necessity for the noise reduction process carried out by the Kalman filter. As such, this research suggests using Kalman filters to enhance state estimates when the market penetration level is below 30%. These findings are consistent with the literature, where Aljamal et al. [57] showed similar findings, where the authors used Kalman filters to estimate the total number of vehicle counts at intersections using probe vehicle data. The results of this paper also confirm the ability of Kalman filters to enhance the estimation of various traffic states such as turning movements, vehicle queues, and traffic stream density.

Compared to previous studies that employed Kalman filters for traffic state estimation, the proposed algorithm in this paper showed superior performance. In regards to turning movement estimation, Emami et al. [25] reported $R^{2}$ values of 0.52 and 0.61 at market penetration levels of 10% and 20%, respectively, while our study reported $R^{2}$ values of 0.70 and 0.82, respectively.

The results of our work also reported comparable queue size estimation accuracy compared to similar recent works in the literature, such as the work of Hu et al., 2022 [30], which reported MAPE values of 39%, 33%, and 23% at market penetration levels of 5%, 10%, and 20%, respectively, while our method reported values of 41%, 33%, and 28% at the same levels of market penetration. While the previously reported performance of queue size estimation is slightly better at low market penetration levels, our method resulted in better performance at higher levels, with MAPE values of 3.3% and 1.4% at MPLs of 80% and 90%, respectively. Moreover, this was reported to be 4.4% and 2.8% at the same levels in the literature, respectively.

Regarding density estimation, previous work by Aljamal et al., 2019 [58] showed similar results in terms of density estimation. Their study reported RMSE results for the tuned adaptive Kalman filter of 32.35 veh/km at a 10% level of market penetration, while our study showed 23.5 veh/km at the same level. Furthermore, our study demonstrated better estimation performance at higher levels of market penetration, reporting RMSE values less than 5 veh/km at MPLs of 80% and higher, while Aljamal’s study reported values of 17.6 and 14.7 veh/km at market penetration levels of 80% and 90%, respectively.

Furthermore, this research addresses the limitations of traffic state estimation and prediction techniques found in the literature using probe data, such as machine learning techniques [59]. Machine learning models typically require historical traffic data and extensive computational resources for training, and they are prone to over-fitting, which compromises their predictive accuracy. The two-stage approach proposed in this research demonstrates enhanced estimation and prediction accuracy without the need for historical traffic data. Furthermore, it exhibits computational efficiency suitable for real-time applications. This research also advances the literature on employing Kalman filters in short-term and real-time traffic state estimation without the need for historical traffic data, which may not be available and may vary temporally.

The presented prediction capability is considered a useful prediction tool for integration into advanced traffic signal control systems, such as the adaptive traffic signal controller proposed by Abdelghafar et al. [2], which requires real-time predictions of TM counts, queue sizes, as well as traffic density estimates for each TM at each approach. Due to the lack of this information in previous research, the proposed system was only tested considering market penetration levels of 100%. However, the assumed high level of market penetration is inconsistent with the current penetration trends, which makes this system inapplicable in the field. The two-stage approach proposed in this research fills the gap in previous research to develop the adaptive game-theoretic system, which requires real-time traffic state estimation and prediction. The availability of this information allows for further deployment and testing of its efficiency at low levels of market penetration. Furthermore, the queue estimation performance shows its efficiency in being integrated into vehicle trajectory optimization systems, in which the real-time position of the back of the queue is essential for vehicle trajectory optimization in the vicinity of traffic signals [60,61].

## 6. Conclusions and Future Directions

This paper introduces a two-stage approach that leverages probe vehicle and upstream detector data for real-time traffic state estimation and prediction. A Kalman filter approach is utilized to provide enhanced traffic state estimates compared to those provided solely by probe vehicle data. In the first stage, the number of turning vehicles is estimated in real-time, and in the second stage, the upstream link density and the number of queued vehicles at the intersection, categorized by movement, are estimated. A sampling error analysis is utilized to estimate the error covariance matrices for the Kalman filter measurements and predictions using real-time data. The proposed system is evaluated using a drone-collected dataset at a four-legged signalized intersection in Orlando, Florida.

Implementation results show that the proposed methodology has demonstrated promising results in enhancing traffic state estimation and prediction capabilities, in terms of turning movements, queue size, and upstream link density. In the first stage, the TM estimation results show a reduction in the error SD of up to 50% relative to the baseline method relying solely on probe vehicle data. The $R^{2}$ of the TM estimates is significantly improved by using Kalman filters from -0.14 to 0.70 at a market penetration of 5%. Similarly, at a market penetration of 10%, the $R^{2}$ improved from 0.27 to 0.82. Furthermore, the first stage showed reliable prediction capability with $R^{2}$ values of 0.68, 0.79, and 0.94 for market penetration levels of 5%, 10%, and 20%, respectively.

In the second stage, the queue size and upstream link density are estimated using Kalman filters utilizing the TM estimates provided in the first stage and probe vehicle data. The results of the queue size estimation showed enhanced estimation accuracy at low levels of market penetration. Comparing the estimated queue size with the actual size, the MAPE was enhanced with up to 45%, with a MAPE value of 41% at a 5% level of market penetration. In addition, the second stage filter produced enhanced estimates of upstream traffic density of up to 18.5%. The proposed approach also shows enhanced estimation and prediction performance at a low level of market penetration.

The research indicates its applicability for field implementation and integration with real-time game-theoretic traffic signal control systems, even at low levels of market penetration. This approach addresses the limitations of existing techniques, such as machine learning models, by providing enhanced accuracy without the need for historical traffic data and with computational efficiency suitable for real-time applications. Moreover, the prediction capability of this algorithm presents a valuable tool for integration into vehicle trajectory optimization systems. Overall, this research fills a crucial gap in previous studies and paves the way for the development and testing of efficient systems at various levels of market penetration, ultimately contributing to the advancement of traffic management and optimization.

Future directions to address the study limitations by enhancing the proposed system by integrating a more sophisticated approach for the market penetration rate estimation, rather than the simple method currently used in the system, as well as including additional data sources at various study locations. Furthermore, future directions also include the integration of the proposed KF approach within an adaptive game-theoretic traffic signal controller; as such, categorized traffic state estimates can be provided to the controller at various market penetration conditions. Therefore, the controller should be suitable for field implementation.

## Figures and Tables

**Table 1:** Intersection demand rate in vehicles/hr according to the drone-based vehicle trajectories.

Approach	LT	THR	RT
NB	71	721	88
SB	188	806	100
EB	121	1223	134
WB	86	844	278

**Table 2:** A comparison between the SD of TMs (vehicles/h) derived from probe vehicle data and using Kalman filtering.

Approach	Market Penetration Levels		
	**5%**	**10%**	**20%**
SD of probe vehicle-estimated TMs	174.1	138.8	56.1
SD of Kalman filter-estimated TMs	90.5	69.1	35.9
Estimation improvement	48.0%	50.1%	36.0%
SD of Kalman filter-predicted TMs	92.8	72.5	42.7

## Data Availability

The original data presented in the study are openly available in UCF-SST-CitySim1-Dataset https://github.com/UCF-SST-Lab/UCF-SST-CitySim1-Dataset (accessed on 28 January 2025).

## Abbreviations

The following abbreviations are used in this manuscript:

TSC	Traffic Signal Control
DNB	Decentralized Nash Bargaining
VOC	Volume-Capacity Ratio
CV	Connected Vehicle
SD	Standard Deviation
OD	Origin-Destination
ANN	Artificial Neural Networks
ATMIS	Automatic TM Identification System
MP	Market Penetration
KF	Kalman Filter
EB	East-bound
NB	North-bound
MAPE	Mean Absolute Percentage Error
